# Prevalence of Transfusion Transmitted Infections and the Quality of Life in β-thalassemia Major Patients

**DOI:** 10.7759/cureus.6129

**Published:** 2019-11-12

**Authors:** May Al-Moshary, Eman Al-Mussaed, Adnan Khan

**Affiliations:** 1 Pathology, Princess Nourah Bint Abdul Rahman University, Riyadh, SAU; 2 Pediatrics, Rehman Medical Institute, Peshawar, PAK

**Keywords:** transfusion transmittable infections (tti’s), hepatitis, tranqol

## Abstract

Objectives

To determine the prevalence of hepatitis B, hepatitis C, and human immunodeficiency virus (HIV) in chronically transfused β-thalassemia major (TM) patients, and to assess their quality of life (QoL).

Methods

This cross-sectional study was conducted in three different thalassemia centers located in Peshawar, Khyber Pakhtunkhwa from January to July 2019. These centers provide screened blood and essential medical care for thalassemia patients. These centers include the Fatimid Foundation, Hamza Foundation, and Rehman Medical Institute, Peshawar, Khyber Pakhtunkhwa. A total of 431 blood transfusion-dependent β-thalassemia patients registered at these centers were selected. QoL in β-TM patients was assessed by a newly developed instrument, the TranQoL questionnaire. For the data analysis procedure, Microsoft Excel and Statistical Package for the Social Sciences; version 22 (SPSS Inc., Chicago, IL) was used.

Results

A total of 431 patients were included in our study. The ages ranged from five years to 23 years with a mean age of 11.54 ± 3.6 years; 58.93% were male and the rest were female with a male to female ratio of 1.43:1. A total of 129 (29.93%) patients were infected by transfusion-transmitted infections (TTIs). Hepatitis C virus (HCV) was found prevalent in 23.66%, hepatitis B virus (HBV) was found in 4.87%, and HIV was found prevalent in 1.39% cases. The results showed a high proportion of HCV in males 27.95% as compared to females 17.51% (p value = 0.31). Patients were divided into high (good) QoL score of >50 and low (poor) score of <50. In patients with hepatitis C, the QoL was poor in 90 (88.23%) patients and was good in only 12 (11.76%) patients (p value=0.01); in the hepatitis B group, it was good in only eight (38.09%) and poor in 13 (61.90%) patients (p-value 0.04), and for patients with HIV, it was poor in all six patients (p=0.001).

Conclusion

Our study concludes that transfusion-transmitted disease is very high and that HCV is the leading TTI followed by HBV and HIV. QoL in patients with TTIs was poor. The use of advanced technology in blood screening, voluntary donations, donor selection, and asepsis during blood transfusion is imperative to curtail the transmission.

## Introduction

Thalassemia is a group of hereditary hemolytic disease caused by decreased or absent production of the alpha or beta-globin chain. Thalassemia is the most common form of single gene disarray. It is approximated that 1.5% of the population around the world has β-thalassemia trait [1]. In Pakistan, the carrier frequency of β-thalassemia is nearly 5%-7% and in general 4,000 to 9,000 cases are added yearly [2]. Routine blood transfusions and iron chelation are a standard of care in thalassemia, and the life expectancy of patients have elevated incredibly [3-4].

During the main treatment in β-thalassemia, major blood transfusion therapy at routine intervals depends upon the nature of the mutation. Blood transfusion at regular intervals leads to certain complexities like iron overload and transfusion mediated infection in thalassemia patients [5]. In developing countries like Pakistan, the most common blood transmitted infection are hepatitis B, hepatitis C, human immunodeficiency virus (HIV), syphilis, and malaria [6].

Regarding hepatitis B and C, Pakistan is viewed as a high prevalence area and its infection is on a steady rise along with β-thalassemia, therefore, it is essential to collect the data of transfusion-transmitted infections (TTIs) in β-thalassemia major (TM) patients [7]. The thalassemia gene is largely restricted to certain families of Pakistan. The children in Pakistan are said to have the highest incidence of β-thalassemia in the world. This might be due to the norms of our system and a higher incidence of consanguineous marriages [8]. TTIs are a major problem in developing countries like Pakistan where blood safety measures are not fully developed. According to the World health organization (WHO), after Egypt, Pakistan has the highest prevalence of hepatitis C in the world [9]. In order to assess the standard of life in thalassemia patients, a biopsychosocial model has been introduced in medicine [10]. Various tools of the biopsychosocial model need to estimate the physical, social and emotional well-being of the patient. These carefully organized questionnaires attempt to quantify these aspects in the patient’s life and accordingly provide us with reproducible data that determine the sequel of thalassemia management and estimate its burden on the loves of patients.

For TM patients, not only is survival important but so is the physical, psychological, emotional, and social functioning as well. This study will also help in assessing the impact of transfusions on the quality of life (QoL) of β-TM patients.

## Materials and methods

This cross-sectional study was conducted in three different thalassemia centers located in Peshawar, Khyber Pakhtunkhwa from January to July 2019. These centers provide screened blood and essential medical care for thalassemia patients. These centers include Fatimid Foundation, Hamza Foundation, and Rehman medical institute, Peshawar. A total of 431 blood transfusion-dependent β-thalassemia patients registered at these centers were selected. Patients with other coexisting hemoglobinopathies were excluded from the study. Information regarding the frequency of transfusion, any other infection and family history were collected by using Performa. All the participants were given an oral and written explanation about the study, including its procedures and were asked to read and sign an informed consent document. The study protocol and ethical aspects were approved by the ethics committee of the Rehman Medical Institute.

Tests for HIV, hepatitis B virus (HBV), hepatitis C virus (HCV) are done by enzyme-linked immunosorbent assay (ELISA) on microplate reader (Biotek ELx 800; Winooski, VT) with washer (ELX50; BioTek Instruments, USA). The HIV status was detected by the microwell ELISA test (Synbiotics Corporation, San Diego, CA) for the detection of antibodies to HIV-1 and HIV-2 in human serum/plasma. HBV status is detected by Qualisa or HEPALISA (Microwell Enzyme Immunoassay, ELISA for the detection of hepatitis B surface antigen (HBsAg) in human serum or plasma). HCV status is detected by HCV-Microlisa (microwell ELISA test for the detection of antibodies to HCV in human serum/plasma).

QoL in beta-TM patients was assessed by a newly developed instrument, the TranQoL questionnaire. This is a specific QoL questionnaire for patients with thalassemia requiring regular transfusion. TranQol assesses patients’ QoL in four domains: physical health, mental health, family health, and career and school function, and comprise 28 questions. This is a self-administered tool with written instructions. The responses are recorded as one of the five options: ‘never’, ‘almost never’, ‘sometimes’, ‘often’, and ‘always’ and coded as 1 to 5 respectively. The questionnaire bears high reliability and constructs validity [11]. The minimum score for all variable questions on tranQol is 28 and the maximum score is 140. Reverse coding was done for negatively phrased questions. In the current study, an interviewer assisted the patients and translated items for the patients with limited literacy ability. Before going for the data collection process, an authorization letter was obtained from the respective authority for the particular place of collection of samples. The confidentiality of scoring/rating to individuals was ensured.

For the data analysis procedure, Statistical Package for the Social Sciences; version 22 (SPSS Inc., Chicago, IL) was used. The mean and standard deviation were calculated for continuous data' whereas, frequency and percentages were calculated for categorical data. A p-value of <0.05 was considered significant. Multivariable analysis of the TranQol was carried out whereby the mean and standard deviation of each variable (item analysis) and that of individual domains (domain analysis) were calculated. To find out the association between TranQol and different clinical parameters, the quantitative variables were converted to categorical variables and Chi-square test was applied to find out its association.

## Results

A total of 431 patients were included in our study. The age of patients ranged from five years to 23 years with a mean age of 11.54 ± 3.6 years. A total of 254 (58.93%) were male and the rest were female with a male to female ratio of 1.43:1. Demographic data are given in Table 1.

**Table 1:** Demographic data of patients (n=431)

Demographic Data	f(%)
Gender	
Male	254(58.93%)
Female	177(41.06%)
Age:	
05-10 years	93(21.57%)
11-15 years	133(30.85%)
16-20 years	117(27.14%)
>20 years	88(20.41%)
Age of onset of Thalassemia	
<06 months	158 (36.65%)
06-5 years	273 (63.34%)
Frequency of Blood Transfusion	
Every 3 week	312(72.38%)
> 4 weeks	119(27.61%)
Splenectomy:	
Yes	241(55.91%)
No	190(44.09%)

**Figure 1:**
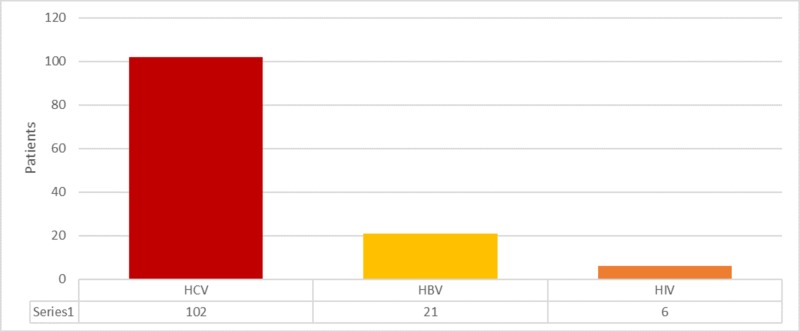
Frequency of transfusion transmitted diseases HCV: hepatitis C virus; HBV: hepatitis B virus; HIV: human immunodeficiency virus.

Among the total 431 patients, 129 (29.93%) were infected with TTIs. Of the 129 TTIs infected β-thalassemia cases, HCV was found prevalent in 102 (23.66%), HBV was found in 21 (4.87%), and HIV was found prevalent in six (1.39%) cases (Figure 1).

The results showed a high proportion of HCV in males 71 (27.95%) compared to the female 31 (17.51%) patients; however, this difference was not statistically significant (p value = 0.31). Similarly, a higher prevalence of HBV was found in males 13 (5.11%) than females eight (4.51%): however, this was also statistically not significant (p value = 0.91). HIV was found more prevalent in females five (1.96%) than in males one (0.56%); however, this difference was also not significant (p value = 0.47) (Table 2).

**Table 2:** Gender distribution of transfusion-transmitted infections (TTIs) in beta-thalassemia patients

	Male (n=254)	Female (n=177)	P value
Hepatitis C	71(27.95%)	31(17.51%)	0.31
Hepatitis B	13(5.11%)	8(4.51%)	0.91
HIV	1(0.56%)	5(1.96%)	0.47

The associations of QoL scores with TTIs mentioned in Table 3 were analyzed. Patients were divided into high (good) QoL score >50 and low (poor) score <50. In patients with hepatitis C, the QoL was poor in 90 (88.23 patients and was good in only 12 (11.76%) patients (p value=0.01), in hepatitis B it was good in only eight (38.09%) and poor in 13 (61.90%) (p-value 0.04), and in patients with HIV, it was present in all six patients (p=0.001).

**Table 3:** Association of transfusion-transmitted diseases with quality of life

	Tran-QOL >50	Tran-QOL <50	P-value
Hepatitis C (n=102)	12 (11.76%)	90(88.23%)	0.01
Hepatitis B (n=21)	8(38.09%)	13(61.90%)	0.04
HIV (n=6)	00	6(100)	0.001

## Discussion

β-thalassemia is a group of hereditary hemolytic disease in which patients require routine transfusing in order to sustain life. In these patients, there is an increased risk of transmission of infection because of regular blood transfusion. Infection disseminated through transfusion has been major complication β-thalassemia patients in developing countries because of the inadequacy of awareness and poor screening practices [6]. Management of patients suffering from β-thalassemia is based on sufficient and safe blood transfusions and receiving routine iron-chelation therapy, all of them improve the QoL as well as the survival of patients. Infections that are mediated by transfusion like HBV, HCV, and HIV are dreaded outcomes of blood transfusions, as these can end in enduring morbidity and mortality. The chief difficulties are because of the high prevalence of asymptomatic carriers in the society, blood donations during the window period of infections, hiding medical history by the captive, paid, or professional blood donors who extensively living in developing countries.

In our study, the mean age of patients was 11.54 ± 3.6 years. Previous studies showed similar figures; a study done by Harfouche et al. [12] showed an average age of 11.5 ± 5.2 in β-thalassemia patients. Furthermore, the study by Ansari et al. [13] approximated the average age as 8.5 ± 6.42 years. The ages of β-thalassemia patients are proportionate to our study findings since a greater number of them are diagnosed at a younger age.

HCV prevalence was found to be 23.66% in the present study, making it the most common TTI; the majority of infected patients were male. It was reported in several local studies between 21%-30% HCV positive cases in β-thalassemia [14-15]. However, some studies report up to 42% [16-17]. These findings of different studies are consistent with our research and revealed that the occurrence of HCV infection is higher than other infectious diseases in patients with β-thalassaemia. In our study, the prevalence of HBV infection was 3.0%, several studies did support our findings, from 1.5%- 2.4% cases of HBV [15,18]. There is scientific evidence that contradicts with our results; Premawardhana et al. reported 6.4% cases of HBV [19]. The studies revealed great variability in findings which is because of the difference in the prevalence of HBV in different parts of the world. In this study, occurrence of HIV in β-thalassemia patients was approximated at 0.5%. According to the majority of studies done from similar sociodemographic countries, HIV has been reported negative [20], nevertheless, this is one of the few studies reporting HIV from Pakistan. Various studies done in various countries have revealed variable findings regarding HIV prevalence; Oza et al. reported 3.1% [21], de Paula et al. reported 17% [22]. In our study, patients were transfused with blood that has been screened for bacterial, viral and parasitic infections. These findings showed that poorly screened blood is the root cause of HCV infection in patients with thalassemia. To minimize the window period, nucleic acid testing is a very sensitive technique but it is not available in a developing country like Pakistan.

This study aimed to measure emotional, physical and social life aspects and overall QoL scores for thalassemia patients with TTIs. Our study reported that Pakistani transfusion-dependent thalassemia children have significantly poor physical QoL compared to international [11] and regional cohorts [23-24]. The majority of the patient in our study with HCV showed poor QoL, with a TranQol score of less than 50. QoL studies have reported lower scores in individuals with thalassemia compared with the general population, especially in individuals with transfusion-dependent thalassemia and having TTIs [25]. Ansari et al. [26] reported in his study that those patients who are transfusion-dependent and have hepatitis C, their QoL is lower. On the other hand, 61.90% of patients with hepatitis B have a TranQol score of less than 50. A study done by Karacaer et al. [27] showed that patients who have HBV have poor QoL scores when the disease is active. Our study showed that all patients with HIV have a poor QoL as their score was less than 50.

## Conclusions

Our study concludes that transfusion-transmitted disease is very high and that HCV is the leading TTI followed by HBV and HIV. QoL in patients with TTIs was poor. The use of advanced technology in blood screening, voluntary donations, donor selection, asepsis during blood transfusion is imperative in curtailing the transmission. Even in developing countries, direct emphasis and appropriate steps should be taken to improve the standard of living and quality of life in patients with β-thalassemia.
